# Higher Education and the Ethic of Care: Finding a Way Forward During a Global Pandemic

**DOI:** 10.1177/15327086211002776

**Published:** 2021-06

**Authors:** Julia Persky

**Affiliations:** 1 Texas A&M University-Commerce, USA

**Keywords:** poetic inquiry, Ethic of Care, higher education, global pandemic, COVID-19

## Abstract

At the outset of the coronavirus pandemic, before we had a chance to acclimate to our world turned upside-down, I was genuinely confounded by the inflexibility of some professors to help students. After all, we had been offered the grace of an extension on tenure-track requirements, and afforded the opportunity, and relative safety, of working from home. How was it possible then, to not understand that students, too, might need grace? And how could anyone be OK with choosing not to help? Far from traditional research questions, my wonderings provoked lively conversations with colleagues, and a lens through which to consider my own positionality and difficulties in dealing with the challenges posed by the pandemic. Framed by Nel Noddings’ Ethic of Care, and through poetic inquiry, this article presents a personal response to teaching in an Educator Preparation Program during a global pandemic.

## Introduction

*I can almost smell the sulfur and brimstone burning in the surrealistic hellscape that has been the year, 2020. Those first reports of the coronavirus in the United States feel like a lifetime ago, and only yesterday. It didn’t take long for media outlets to question what a global pandemic would mean for Higher Education, for tenure-track faculty, for staff, for students who lived in campus housing, for sports, for academics.*

*Moving the entire University online meant many days where even finding time to take a breath was a challenge. Once-a-month faculty meetings turned into weekly Zoom meetings, as we worked to keep our Educator Preparation Program coordinated and running smoothly, across multiple campus locations. On top of all the other disruptions and complications, we had student teachers in the field for clinical teaching required by the state, in addition to students enrolled in courses that required classroom observation hours. It took weeks to receive information from the Texas Education Agency as acceptable means of meeting state requirements shifted repeatedly. Even with an extra week of spring break, there was no way to anticipate, or prepare for, the challenges we would face in serving our students. By the time we resumed classes, many of our students were already faced with staggering, life-altering, consequences of the coronavirus, including job loss or furlough, work from home, homeschooling, daycare closures, and food scarcity.*

*It was no surprise when emails requesting deadline extensions or assistance with assignments due to lack of internet access or technology began rolling in. I was surprised, however, when we began to receive emails from campus administration—President, Provost, Dean, Department Chair—requesting that we please be flexible with students, help them, support them any way possible. Weren’t we already doing that? I wondered. Yet, over and over we received those emails, until finally it was addressed in one of our department faculty meetings. Apparently, there were some faculty members who were less inclined than others, to work with students in a flexible manner. I found this extremely frustrating; in part because it wasn’t fair to students, and in part because it made more work for others, and we were all already stretched thin.*

*During a phone conversation with a colleague, I expressed my dismay, explained that in some other department or program, maybe it was, to some degree, understandable—but in our department? “How is it OK?” I asked her. “How is it OK to not help?! To not care?! We’re TEACHERS! Every single one of us are teachers. We’re supposed to be the caregivers. How can we expect our students (future teachers) to demonstrate empathy for their students, or offer compassion to the families they will serve, if we don’t model it for them?” Her reply saddened me, “Julie, I’m not so sure that everyone in the department sees themselves as caregivers, or perceives that caring is part of their role. They see themselves as professors/instructors, with information to disseminate, and the rest is up to the student.” With that explanation, I felt some things click into place, and a clearer image took shape for me. COVID-19 has shown us the callousness of some among us, but it’s also shown us our courage, our compassion, and our capacity for doing good. To my way of thinking, COVID-19 has offered us that rare glimpse into the immediate consequences our choices have on the lives of others. I understand we all have different approaches to teaching, and reasons for doing the things we do in our classes, and for me, I knew the only way I would have peace within myself, was to approach teaching through the Ethic of Care.*

## Methodology and Theoretical Framework

At the outset of the coronavirus pandemic, before we had a chance to acclimate to our world turned upside-down, I was genuinely confounded by the inflexibility of some professors to help students. After all, we had been offered the grace of an extension on tenure-track requirements, and afforded the opportunity, and relative safety, of working from home. How was it possible then, to not understand that students, too, might need grace? And how could anyone be OK with choosing not to help? Far from traditional research questions, my wonderings provoked lively conversations with colleagues, and a lens through which to consider my own positionality and difficulties in dealing with the challenges posed by the pandemic.

Framed by Nel Noddings’ Ethic of Care, this article presents a personal response to teaching during a global pandemic. Noddings (2005) established the Ethic of Care as foundational to human morality. At its most basic, the care ethic is a needs- and response-based ethic, dependent upon a relationship between at least two people, the carer and the recipient of care, or cared-for. Unlike other ethics theories, the Ethic of Care rejects the principle of universality, as caring actions are based on the needs of a unique person within the context of unique circumstances.

For this project, poetic inquiry served as method of analysis. Poetic inquiry made room for poems “. . . crafted as project analysis, and/or poems that are part of or constitute an entire research project” (Faulkner, 2016, p. 20). Poetic inquiry lent itself well to this article due to poetry’s evocative use of language that engages the cognitive and the sensory (Faulkner, 2016; Leavy, 2015; Richardson, 1997). Thus, I was able to adapt stories my students shared as representative of their experiences, but also, to simultaneously wind and unwind their stories with my own, as I too, grappled with interpreting and understanding (Brady, 2004) personal experiences related to teaching in a global pandemic, over the past year.

### Fall: Finding a Way Forward

When the decision was made to move most of our courses online for the fall, work commenced on the redesign of face-to-face courses for online instruction. For one course alone, six of us collaborated, throughout the summer, to make the course engaging, to ensure the assignments were of high quality, with rigor appropriate for the content, and to align activities with the Student Learning Outcomes. We critiqued and refined, and refined, and refined. And still, once the fall semester began, we were inundated with challenges.

Our Educator Preparation Program (EPP) is offered on four campuses, the main campus, and three Extended University locations, so courses are aligned across campuses to ensure students receive the same standard of learning no matter which campus they attend. However, the four programs are located in areas with vastly different student demographics: two rural campuses, one suburban campus, and one urban campus. The students in each location have varying degrees of access to resources.

Based on emails I received from a large number of students, I knew there would be no single path forward for us all—every student would need support in ways that met their unique circumstances, what worked for one would not work for all (Noddings, 2012), and what worked on one campus would not work across all of our locations. In spite of all our planning, we were, in many ways, on our own. I would have to be involved with each student personally, ascertain their need and devise a plan with them, that enabled them to achieve success in their courses while also attending to the more immediate needs of home and family (Noddings, 2005, 2012).

Although I tried, there was just no way I could have my students complete the assignments in the same ways as students at other locations, as the majority of them did not have reliable internet access, and many of them did not have personal devices other than their phones, on which to complete coursework. The challenges my students faced could not be mitigated via flashy new tech gizmos, or digital media, or technology at all, really. They were challenges that required critical thinking and problem solving, challenges that required personal investment of time, energy, and care (Noddings, 2012), and there isn’t an app for that.

For my students, the choices and accommodations made to support them not only made a difference in how they experienced school (Nieto & Bode, 2008), for some of them, it meant the difference in whether they continued their degree program or dropped out of school. The Ethic of Care meant finding a way for them to stay on track, finding ways to ensure, as much as possible, an equal opportunity of outcomes, it meant doing no harm academically (Noddings, 2012).

### How We Go to School

Please share -

The survey asked,

Your internet and technology

Accessibility

One by one

They each replied

Not one of them

Complained

When I get off work,

I take my 3 sisters to

Starbucks

We use their internet

From the parking lot

After they close

I go to

Sonic

And park in

Back

We go to the

School

And use their

Wi-Fi

From the

Parking lot

I go to my

Friend’s

House

I use the internet

At my

Mom’s

Job

My neighbor lets me

Use her computer

And her internet

When my

Phone

Runs out of

Data

They were doing

Their

Best

And not one

Of them

Complained.

**Heroes**

Student Teachers

In the field

Faced harsh

Reality

Learned

Early on

Their needs

Concerns and

Health are

Low priority

“This school –

they aren’t

even

TRYING

To follow

Protocols!”

“A kid gets sick.

They send them home.

Close the door.

And say,

‘Do not speak of this.’”

And

“At my school –

parents yell at us –

at my mentor and at me”

“They don’t want

their child to wear a

Mask.

It’s not the way

Their kid should

Go to school,

They say.”

“I know I have to be here

But I don’t know what to do.

What if I get sick?

What about my own family?”

### Breakfast

No one has eggs!

  Or milk.

  I went to

 3 grocery stores,

  looking for enough food to feed my family of

 6 people.

No one has eggs!

  Or milk.

  I have

 4 kids,

  and I made

 3 breakfasts

  this morning, because I could not buy enough of anything to make

 1 meal

No one has eggs!

  Or milk.

  When I did find eggs, I could only buy

 1 carton,

  but not the carton with

 12 eggs.

  it was one of those little cartons, with

 6 eggs,

  because the store limited purchases to

 1 carton.

  And the cashier was dumb, and wouldn’t let me buy

 2 cartons

  so I could have

 1 dozen.

  I have

 4 kids,

  and I made

 3 breakfasts this morning.

No one has eggs!

  Or milk.

### Pandemic Pleas

“Dr. Persky,

my assignment is late . . .”

“My husband has been in the hospital with COVID.

I have health issues of my own. And we have

 Four kids that I’m currently homeschooling.”

“My whole family has COVID, and

I’m in quarantine with my grandparents.

 They don’t have the Internet.”

“My Godmother had COVID, and I’ve been

 out of town so I could be with her

  before she died.”

“My twins were exposed to COVID at their daycare.

I’m home with them for the next two weeks.

 I’ll do my best to keep up and let you know

  If I get sick.”

“My school, where I work, called me tonight, to tell me –

I’ve been directly exposed to COVID, by a student in the class.

 My first test was negative.

Four days later, my second test was positive.

 I’m in the hospital now, unable to keep up with coursework.

Finally, I’m home and doing better, though it’s still hard for me to walk.

 I’ll get caught up as fast as I can.”

“Dr. Persky,

My husband died.

 We’ve been together since we were 14 years old.

  We have four kids.

  I have health issues of my own.

  It’s hard to breathe. I am so lost.

Dr. Persky,

  My husband died.”

## Concluding Thoughts

I began this article with a personal reflection regarding my experience as a faculty member, in the early days of the coronavirus pandemic, and the realization that there was no way to plan for many of the situations that have presented themselves over the course of the last year. Indeed, no amount of planning could have prepared us for the upheaval and trauma that many of our students, faculty, and staff have endured. As an academic advisor, field supervisor, and assistant professor, my path through the uncertainty and chaos has been the Ethic of Care (Noddings, 2012). Engaging with students individually enabled me to provide meaningful and effective support based on each unique circumstance, and poetry provided the space to feel, and to cope with our collective pain. The Spring 2021 semester is upon us, and we are all weary. As COVID-19 continues to ravage the globe, and continued uncertainty looms on the horizon, my hope is we might each be reminded that we all need help sometimes, and lead with care.
